# Treprostinil reduces endothelial damage in murine sinusoidal obstruction syndrome

**DOI:** 10.1007/s00109-018-1726-6

**Published:** 2018-12-07

**Authors:** Madeleine Themanns, Florian Koban, Christian Bergmayr, Alicja Chrzan, Wolfgang Strohmaier, Johannes Haybaeck, Michael Freissmuth, Eva Zebedin-Brandl

**Affiliations:** 1 Institute of Pharmacology and the Gaston H. Glock Research Laboratories for Exploratory Drug Development, Centre of Physiology and Pharmacology, Medical University of Vienna, Vienna, Austria; 2 Department of Pathology, University Hospital and Medical Faculty, Otto von Guericke University Magdeburg, Magdeburg, Germany; 3 Department of Pathology and Laboratory Diagnostics, Maria Sklodowska-Curie Memorial Cancer Centre and Institute of Oncology, Warsaw, Poland; 4 SciPharm SàRL, 2540 Luxembourg City, Luxembourg; 5 Institute of Pathology, Medical University of Graz, Graz, Austria; 6 Department of Pathology, Medical University of Innsbruck, Innsbruck, Austria

**Keywords:** Prostacyclin, VOD, SOS, HSCT, Defibrotide, Transplant-related toxicity

## Abstract

**Abstract:**

Sinusoidal obstruction syndrome (SOS) is a major complication after hematopoietic stem cell transplantation and belongs to a group of diseases increasingly identified as transplant-related systemic endothelial disease. Administration of defibrotide affords some protection against SOS, but the effect is modest. Hence, there is unmet medical need justifying the preclinical search for alternative approaches. Prostaglandins exert protective actions on endothelial cells of various vascular beds. Here, we explored the therapeutic potential of the prostacyclin analog treprostinil to prevent SOS. Treprostinil acts via stimulation of IP, EP_2_, and EP_4_ receptors, which we detected in murine liver sinusoidal endothelial cells (LSECs). Busulfan-induced cell death was reduced when pretreated with treprostinil in vitro. In a murine in vivo model of SOS, concomitantly administered treprostinil caused lower liver weight-to-body weight ratios indicating liver protection. Histopathological changes were scored to assess damage to liver sinusoidal endothelial cells, to hepatocytes, and to the incipient fibrotic reaction. Treprostinil indeed reduced sinusoidal endothelial cell injury, but this did not translate into reduced liver cell necrosis or fibrosis. In summary, our observations provide evidence for a beneficial effect of treprostinil on damage to LSECs but unexpectedly treprostinil was revealed as a double-edged sword in SOS.

**Key messages:**

- Murine liver sinusoidal endothelial cells (LSECs) express prostanoid receptors.
- Treprostinil reduces busulfan-induced cell death in vitro.
- Treprostinil lowers liver weight-to-body weight ratios in mice.
- Treprostinil positively affects LSECs in mice but not hepatic necrosis/fibrosis.

## Introduction

Sinusoidal obstruction syndrome (SOS), previously referred to as veno-occlusive disease (VOD), is a severe hepatic complication that occurs after hematopoietic stem cell transplantation (HSCT). Initial damage of liver sinusoidal endothelial cells (LSECs) caused by the combination of high-dose cytotoxic drug therapy/irradiation and inflammatory mediators released in an allogeneic immune reaction are central to the pathophysiology of the disease [1, 2]. The course and severity of SOS are highly variable and difficult to predict: mild to moderate SOS typically resolves within weeks. In contrast, severe SOS can progress to multi-organ failure rendering SOS a life-threatening orphan disease [3].

Defibrotide is the only drug approved for the treatment of SOS [4, 5]. Its mechanism of action is enigmatic: originally, defibrotide was shown to stimulate the production of prostacyclin/PGI_2_ in different vascular beds [6, 7] and to act as an agonist at A_1_- and A_2A_-adenosine receptors [8], accounting for some of the beneficial actions [9]. Stimulation of G_s_-coupled receptors, such as the A_2A_-adenosine receptor and the β_2_-adrenergic receptor, increases the proliferation and survival of endothelial cells [10, 11]. The cognate receptor of prostacyclin/PGI_2_ is also a G_s_-coupled receptor. Inflammatory cytokines and other mediators reduce endothelial prostacyclin/PGI_2_ production [12]. Conversely, activation of the I prostanoid (IP) receptor and possibly of other G_s_-coupled E prostanoid receptors (EP_2_, EP_4_) elicits protective actions [12]. In addition, IP receptor stimulation counteracts fibrotic stimuli [13, 14]. In fact, when prophylactically administered by continuous intravenous infusion, PGE_1_ halved the incidence of SOS after allogeneic bone marrow transplantation [15]. However, an independent trial failed to replicate the beneficial effect [16]. In contrast to PGE_1_, treprostinil is selective for G_s_-coupled receptors with a preference of IP receptors [17]. Treprostinil was approved for the treatment of pulmonary hypertension. Accordingly, the clinical experience with treprostinil covers the sum of several thousand patient years: treprostinil is reasonably well tolerated and its human pharmacology is well understood [18–21].

Here, we explored the hypothesis that treprostinil interfered with the cascade triggering and/or sustaining damage to LSECs in a murine model of SOS. We examined the action of treprostinil in mice subjected to allogeneic hematopoietic stem cell transplantation. The observations provided evidence for a beneficial effect of treprostinil on damage to LSECs, but this failed to translate into precluding the development of SOS.

## Materials and methods

### Isolation of primary murine cells and experiments with transformed murine LSECs

Primary hepatocytes and LSECs were isolated by liver perfusion as described previously [22]. Isolated primary hepatocytes and LSECs were immediately homogenized for RNA extraction. Vijay H. Shah (Mayo Clinic and Foundation, Rochester, MN, USA) kindly provided transformed murine sinusoidal endothelial cells (TSECs) with stable expression of SV40 large T-antigen [23]. TSECs were cultured in endothelial cell medium containing 5% fetal bovine serum, 1% penicillin/streptomycin, and 1% endothelial cell growth supplement (ECGS) (ScienCell Research Laboratories, San Diego, CA). For cell viability experiments, 24 h after plating (5 × 10^3^ cells/24-well plate), 10 μM treprostinil was given to the medium. After 1 h pretreatment with treprostinil, busulfan (Sigma-Aldrich, Vienna, Austria) was added at concentrations ranging from 10 μM to 1 mM. Cell viability was assessed after 48 h. For this purpose, cells were washed twice with PBS, trypsinized, and counted using a hemocytometer and trypan blue. For MTT cell metabolic activity experiments, 24 h after plating (1.5 × 10^3^ cells/96-well plate) in phenol-red free medium, 10 μM treprostinil was given to the medium. After 1 h pretreatment with treprostinil, busulfan was added at concentrations ranging from 125 μM to 1 mM. MTT assay was assessed after 48 h according to manufacturer’s instructions and absorbance was measured at 570 nm (Thermo Fisher Scientific, Waltham, MA). For [^3^H]cAMP accumulation assays, TSECs were incubated for 16 h with medium containing [^3^H]adenine (1 μCi ml^−1^) and samples were processed as described previously [24–26]. Treprostinil (Remodulin®) was kindly provided by SciPharm SàRL (2540 Luxembourg City, Luxembourg).

### Analysis of gene expression by quantitative PCR

Total RNA was isolated with TRI® Reagent (Sigma-Aldrich, Vienna, Austria) according to the manufacturer’s protocol. Total RNA was reverse-transcribed into complementary DNA (cDNA) using High Capacity cDNA Reverse Transcription Kit (Thermo Fisher Scientific, Waltham, MA). The levels of murine transcripts encoding prostanoid I and prostaglandin receptors (*Ep*_1_, *Ep*_2_, *Ep*_3_, *Ep*_4_, and *Ip*) were assessed by quantitative PCR (qPCR). Murine 18S ribosomal RNA (*18s*) was used as a reference gene for qPCR. Each reaction condition was performed in triplicates. Relative abundance was calculated using the 2^–ΔCt^ method (gene-specific expression level relative to that of the reference gene). The specific primer sequences listed in Table 1 were purchased from Microsynth AG (Balgach, Switzerland).

**Table 1:** Primer sequences used for qPCR analysis

Murine gene symbol	Forward primer	Reverse primer
*Ep*_1_	AGCAGGAGCCAAGTTCCAG	CATCCGCTAGGCTCAGGTTA
*Ep*_2_	TTATGACCATCACCTTCGCC	TAAAAACCGAAGAGCTCGGA
*Ep*_3_	TGGATCCCTGGGTTTATCTG	GGGAAACAGGTACTGCAATGA
*Ep*_4_	TCTCTGGTGGTGCTCATCTG	TGCAAATCTGGGTTTCTGCT
*Ip*	GGGCACGAGAGGATGAAGT	GATGGCCTGAGTGAAGCCT
*18s*	GTAACCCGTTGAACCCCATT	CCATCCAATCGGTAGTAGCG

### Mice

Male BALB/c and C57BL/6J mice were either purchased from the Jackson Laboratory (Bar Harbor, ME) through Charles River Germany (Sulzfeld, Germany) or were bred in-house (C57BL/6J). Mice were between 8 and 10 weeks old, and their body weight was in the range of 20–25 g. Animal housing and husbandry were in accordance with the recommendations and requirements defined by the Federation of Laboratory Animal Science Associations (FELASA) in Europe. Mice were kept on a 12-h light-dark cycle in isolated ventilated cages with ≤ 5 mice/cage (Smart Flow and Easy Flow; Tecniplast, Buguggiate, Italy) at 21 ± 3 °C. Mice were fed autoclaved standard laboratory chow (commercial control diet for mice; Ssniff R/M-H, Soest, Germany) and water ad libitum. Animal technicians monitored animal welfare and health status daily under the supervision of a veterinarian. The experimental protocol was reviewed by the animal ethics committee of the Veterinary University of Vienna, approved by the Austrian Ministry of Science and Research under licenses BMWFW-68.205/0103-WF/V/3b/2015 and BMWFW-68.205/0047-V/3b/2018 and conducted according to the guidelines of FELASA and ARRIVE. Predefined humane end points included emaciation (i.e., weight loss > 25%), loss of activity (reduced mobility, prolonged crouching), loss of grooming/ruffled fur, or labored breathing. Mice meeting these criteria were killed by cervical dislocation.

### Experimental animal model

The murine model of SOS described by Zeng et al. [27, 28] is based on an allogeneic hematopoietic stem cell transplantation (allo-HSCT), where preconditioning is achieved by whole body irradiation. In that model, the maximum of SOS is observed 15 days after allogeneic hematopoietic stem cell transplantation. After adaptation for 7 days, BALB/c mice were randomly divided into three groups: healthy control, allo-HSCT, and allo-HSCT + treprostinil. Mice assigned to allo-HSCT + treprostinil were pretreated 1 day before HSCT with treprostinil subcutaneously (0.15 mg kg^−1^ 8 h^−1^) as described previously [24]. On the same day, recipient mice, i.e., the groups allo-HSCT and allo-HSCT + treprostinil, were exposed to whole body irradiation (7.5 Gy, split doses, 2 Gy min^−1^; Siemens Primus, 6MV, Siemens Austria): mice were placed in single chambers of an irradiation pie with 15 mice per pie. The radiation dose delivered was verified with a dosimeter. On the next day, recipient mice received 5 × 10^6^ bone marrow mononuclear cells containing 1.14 ± 0.26% hematopoietic stem cells via tail vein injection. Bone marrow mononuclear cells were obtained from donor C57BL/6 mice. Mice belonging to the group allo-HSCT + treprostinil were daily treated with subcutaneous injections of treprostinil for 15 days. Thereafter, all mice (healthy control, allo-HSCT, allo-HSCT + treprostinil) were euthanized.

### Histology and immunohistochemistry

On day 15 after HSCT, mice were weighed and livers were immediately removed after euthanasia, weighed, fixed in 4% formalin, and embedded in paraffin for subsequent slicing. Liver weight was normalized for body weight by calculating the liver weight/body weight ratio. Sections prepared from formalin-fixed, paraffin-embedded organ specimens were stained with hematoxylin-eosin (H&E) or with Masson-Goldner trichrome using standard protocols. Light microscopic images were captured with a PixeLINK camera and the corresponding acquisition software on a Zeiss Imager Z.1.

### Scoring of histopathology

Histological sections (two sections/animal) stained with H&E and Masson-Goldner trichrome were evaluated by a board-certified pathologist (JH), who was blinded to the nature of the treatment. The scoring system was based on the system described by Qiao and Zeng et al. [27, 28]. Histological changes were reviewed and scored (0 to 3 points/item) for the following seven pathological features: (i) endothelium injury in liver sinusoid or small hepatic veins, (ii) subendothelial hemorrhage, (iii) internal hemorrhage in the hepatic sinusoid, (iv) liver cell necrosis, (v) fibrosis of the central veins, (vi) hepatic sinusoidal fibrosis, and (vii) inflammation in the central veins.

### Blood sampling and evaluation of plasma parameters

Blood of euthanized mice was collected by heart puncture for analysis of blood cell count and biochemical parameters: white blood cell (WBC), red blood cell (RBC), and platelet (PLT) counts were determined using the Vet animal blood counter (scil animal care, Viernheim, Germany). Plasma levels of bilirubin and alanine aminotransferase (ALT) were assessed using the test strip-based Reflotron Plus analyzer (Roche, Basel, Switzerland).

### Statistical analysis

The primary outcome parameter was hepatomegaly, and the number of animals was based on the following assumptions: the liver-to-body weight ratio (liver weight as % of body weight) was to increase from a mean of 5.5% with a standard deviation of 0.5% to 7.5 ± 0.7% on day 15 [27]. This increase was to be halved by treprostinil. Based on this assumption, we calculated that 18 animals/group were required to detect a statistically significant difference of *p* < 0.05 with a 90% probability. We included one and two additional mice in the treprostinil-treated and in the control recipient group, respectively, to account for possible dropouts due to bone marrow transplant failure; treprostinil enhances bone marrow transplantation [24], hence we assumed that the dropout rate would be smaller. Secondary outcomes included ALT, bilirubin, and pathohistological scores.

## Results

### Target receptors of treprostinil are expressed by murine LSECs

**Fig. 1:**
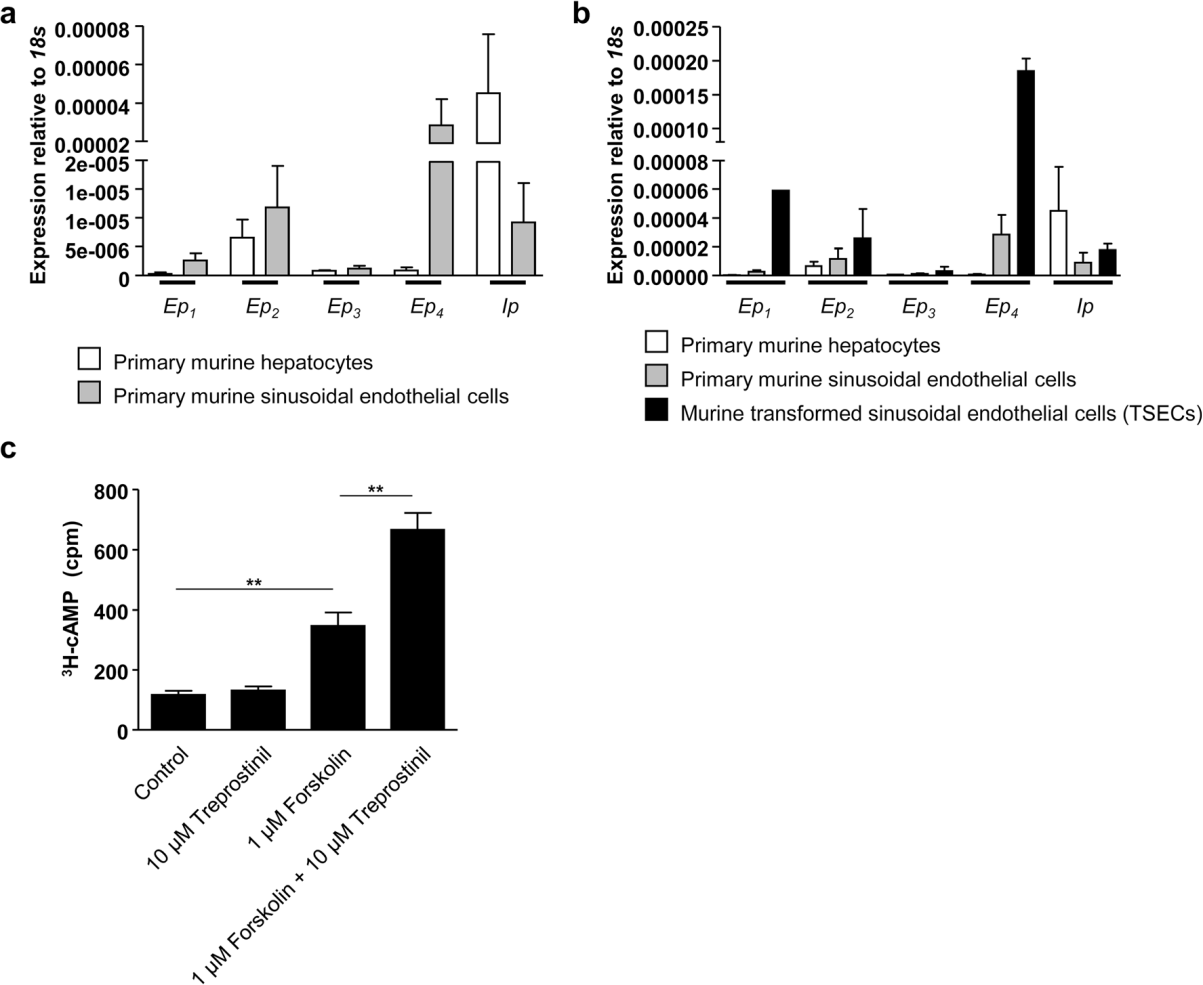
Murine LSECs express target receptors of treprostinil resulting in cAMP accumulation. **a** mRNA was isolated from primary murine LSECs and hepatocytes and reverse-transcribed to cDNA. Expression profile of target receptors of treprostinil was assessed by qPCR-dependent amplification using primers listed in Table 1. mRNA expression levels of E and I prostanoid receptors (*Ep*_1–4_ and *Ip*) were analyzed. Murine *18s* served as reference gene for normalization of qPCR experiments. Data are represented as means ± standard error of the mean. **b** Isolated mRNA from murine transformed sinusoidal endothelial cells (TSECs) was reverse-transcribed to cDNA. Expression profile of target receptors of treprostinil was analyzed by qPCR-dependent amplification using primers listed in Table 1. mRNA expression levels of E and I prostanoid receptors (*Ep*_1–4_ and *Ip*) were analyzed and compared to the expression profiles of primary murine LSECs and hepatocytes. Murine *18s* served as reference gene for normalization of qPCR experiments. Data are represented as means ± standard error of the mean**. c** Murine TSECs were stimulated with 10 μM treprostinil, 1 μM forskolin, or the combination of treprostinil (10 μM) and forskolin (1 μM). Sole addition of treprostinil was not sufficient to increase intracellular cAMP levels in TSECs. Yet, when potentiated by forskolin, treprostinil further increased intracellular cAMP levels. Data are represented as means ± standard error of the mean. Differences between groups were examined for their statistical significance by one-way ANOVA followed by Tukey’s post hoc test for multiple comparisons (**p < 0.01)

Treprostinil, a stable analog of PGI_2_, stimulates IP, EP_2_, and EP_4_ receptors [17]. Our working hypothesis posits that treprostinil protects against SOS by acting on LSECs, the cell type thought to be responsible for the initiation of SOS. We isolated mRNA from primary murine LSECs and hepatocytes and evaluated the expression of E prostanoid receptors by qPCR, i.e., *Ep*_1_, *Ep*_2_, *Ep*_3_, *Ep*_4_, and *Ip*. Amplicons of receptors were detected in both primary murine hepatocytes and LSECs (Fig. 1a). The expression of treprostinil-target receptors *Ep*_2_, *Ep*_4_, and *Ip* was robust in LSECs with exceeding expression levels of the *Ep*_4_ receptor. To reduce animal numbers and circumvent technical difficulties (quantity; maintenance) with primary LSECs, we utilized an immortalized cell line derived from murine LSECs [23]. Similar to primary murine LSECs, these transformed sinusoidal endothelial cells (TSECs) displayed robust expression of all target receptors of treprostinil, with *Ep*_4_ receptor expression being the highest (Fig. 1b). It is difficult to estimate how transcript levels and protein levels correspond, because there are neither good antibodies directed against EP and IP receptors nor suitable commercially available antagonist radioligands. Accordingly, we restored to measuring the cAMP response. Hematopoietic stem and progenitor cells have a comparable expression profile of Gs-coupled E and I prostanoid receptors (EP_2_, EP_4_, and IP). At a concentration of 10 μM, treprostinil produced a robust near maximum stimulation of cAMP accumulation in hematopoietic stem and progenitor cells and its action was potentiated by forskolin [22]. Accordingly, we measured the cAMP response of TSECs in the presence of 10 μM treprostinil, 1 μM forskolin, and the combination thereof. The adenine nucleotide pool of TSECs was metabolically labeled with [^3^H]adenine and their response to treprostinil and forskolin was examined. While sole addition of treprostinil did not suffice to increase intracellular cAMP levels in TSECs (Fig. 1c), cells sensitized with forskolin further increased intracellular cAMP levels in TESCs in the presence of treprostinil. Accordingly, we concluded that TSECs can be used to study the effects of treprostinil in vitro.

### Treprostinil attenuates busulfan-induced cell death in TSECs

**Fig. 2:**
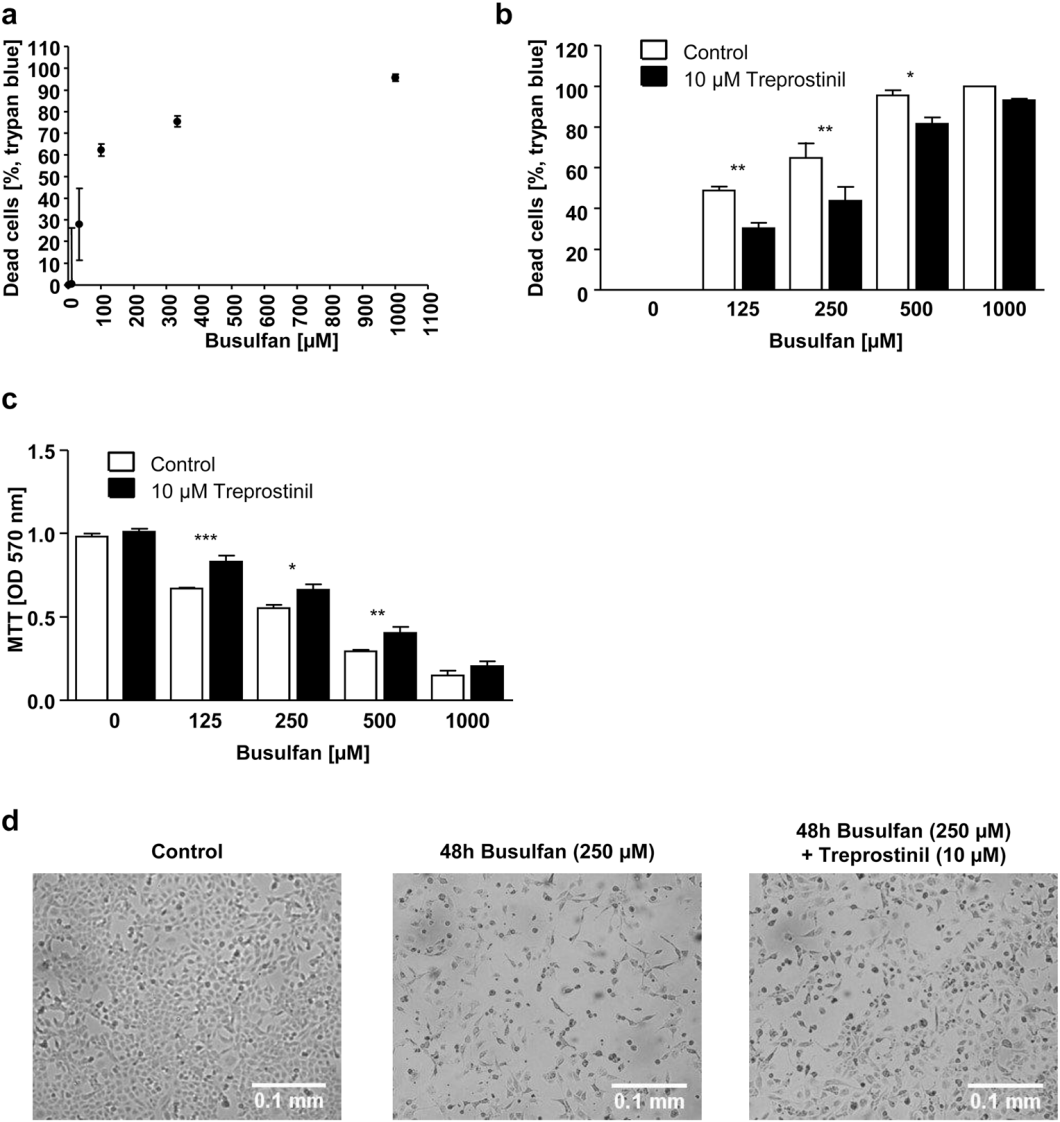
Treprostinil protects TSECs against busulfan-induced cell death. **a** Concentration-response curve for busulfan-induced death of TSECs. Cells were plated and after 24 h busulfan was added at increasing concentrations (10 μM to 1 mM). Cell viability was assessed after 48 h by trypan blue. Data are represented as means ± standard deviation. **b**, **c** Treprostinil protected TSECs against busulfan-induced cell death. Cells were plated and after 24 h treprostinil was added at a concentration of 10 μM. One hour later, busulfan was added at increasing concentrations as indicated. Cell viability was assessed after 48 h by trypan blue exclusion (**b**) and by an MTT assay (**c**). Treatment with treprostinil was associated with increased viability, if the cells were exposed for 48 h to busulfan at increasing concentrations. Data are represented as means ± standard error of the mean. Data was analyzed using one-way ANOVA followed by Bonferroni’s post hoc test for multiple comparisons (*p < 0.05, **p < 0.01, ***p < 0.001). **d** Cell morphology of TSECs after treatment with busulfan for 48 h. TSECs were treated with 250 μM busulfan for 48 h or pretreated for 1 h with 10 μM treprostinil before the addition of busulfan

In clinical conditioning regimes based on high-dosed busulfan, the increased risk of SOS is related to the toxicity of busulfan [29]. To test whether the toxicity of busulfan could per se induce cell death in TSECs, the compound was added at increasing concentrations and cell viability was assessed by trypan blue exclusion after 48 h. Indeed, busulfan induced cell death in a concentration-dependent manner (Fig. 2a). For this reason, we next investigated whether pretreatment with treprostinil and its continuous presence reduced busulfan-induced cell death. Therefore, treprostinil was added to TSECs 1 h before the addition of busulfan. Treprostinil attenuated busulfan-induced toxicity, which was assessed by cell viability, metabolic activity, and cell morphology after 48 h (Fig. 2b–d). Given the positive outcome observed in the in vitro studies, we further elucidated the effect of treprostinil in a living organism.

### Pretreatment with treprostinil reduces hepatomegaly

**Fig. 3:**
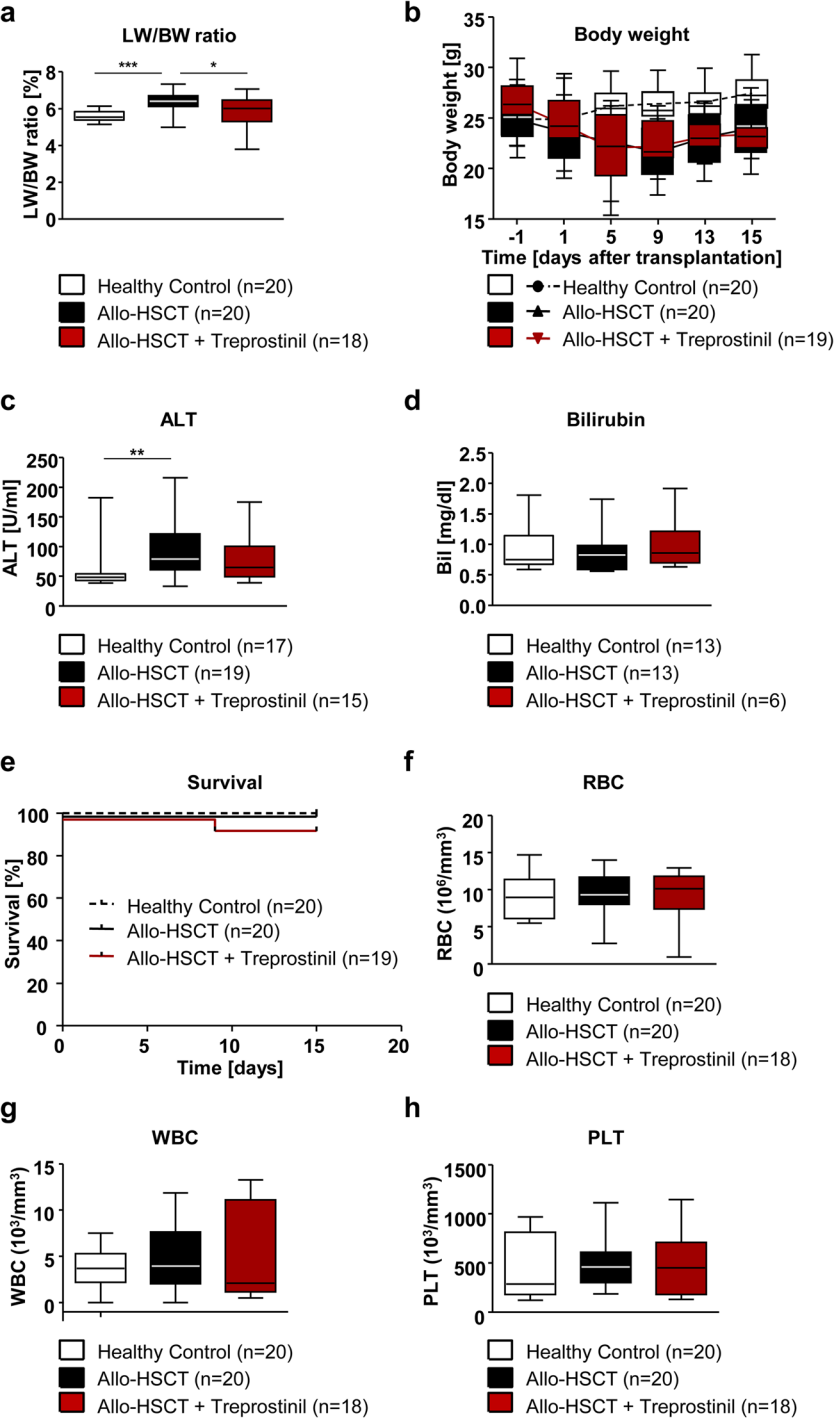
Effect of treprostinil on liver-to-body weight ratio (LW/BW) in mice, which underwent allogeneic hematopoietic stem cell transplantation (allo-HSCT). BALB/c mice were preconditioned with whole body irradiation (7.5 Gy) and received bone marrow cells from C57BL/6 mice via tail vein injection. One group of mice was treated subcutaneously 1 day before and the subsequent 15 days after allo-HSCT with treprostinil (0.15 mg kg^−1^ 8 h^−1^). **a** Mice, which underwent allo-HSCT, suffered from hepatomegaly indicated by LW/BW ratios, but treprostinil-treated mice (allo-HSCT + treprostinil) were protected. **b** Body weight of all mice was assessed by regular measurements at the indicated time points. Note that one mouse undergoing allo-HSCT, which was treated with treprostinil, was euthanized on day 10 (see panel **e**); accordingly, *n* = 18 for the last 2 time points. **c**, **d** Assessment of liver damage parameters. Plasma levels of ALT were significantly increased only in allo-HSCT mice, while bilirubin levels were comparable in all groups. **e** Kaplan-Meier plot of experimental BALB/c mice over a time period of 15 days after allo-HSCT. Kaplan-Meier plot showed no difference in survival. **f**–**h** Blood cell counts 15 days after allo-HSCT. Blood was collected of sacrificed mice and the indicated parameters (WBC = white blood cell count, RBC = red blood cell count, PLT = platelet count) were measured. After allo-HSCT, there were no appreciable differences in blood cell counts between untreated and treprostinil-treated mice. All data are represented as median ± interquartile range with whiskers indicating maximum/minimum range. Differences between groups were examined for their statistical significance by the Kruskal-Wallis test followed by Dunn’s post hoc test for multiple comparisons (*p < 0.05, **p < 0.01, ***p < 0.001)

We examined the action of treprostinil in a murine model of SOS, which relied on allo-HSCT in mice preconditioned by whole body irradiation [27]. Our approach was designed to detect a protective effect on the early insult to the sinusoidal endothelium. Accordingly, irradiated recipient BALB/c mice were subcutaneously treated with treprostinil 1 day prior to and the subsequent 15 days after the allo-HSCT (group: allo-HSCT + treprostinil; 0.15 mg kg^−1^ 8 h^−1^). We selected this time interval based on the report of Zeng et al. (2013), where the signs of SOS reached a maximum on day 15. As the idea here was to facilitate HSCT and prevent SOS within the same treatment regime, we used in vivo the dose of 0.15 mg kg^−1^ 8 h^−1^ treprostinil, which in previous work had been shown to be optimal to improve HSCT [24]. Consistent with previous observations [27], we observed an increase in liver-to-body weight ratio from about 5.5% in the untreated control group to about 7.5% in recipient mice, which had undergone allogeneic hematopoietic stem cell transplantation (Fig. 3a). Administration of treprostinil protected against hepatomegaly: there was a statistically significant difference between untreated recipient mice and those treated with treprostinil (Fig. 3a). This difference in liver-to-body weight ratio was not accounted for by a distinct change in body weight: both untreated and treprostinil-treated recipient mice lost weight over a similar time course, the nadir was reached between days 6 and 9 (Fig. 3b). Similarly, we also determined the levels of circulating ALT (Fig. 3c) and bilirubin (Fig. 3d): the enzymatic marker of liver cell damage was elevated in recipient mice, when compared to control animals (Fig. 3b), but there was no statistically significant difference between untreated and treprostinil-treated recipient mice (Fig. 3b). In contrast, bilirubin levels were comparable in all three groups (Fig. 3c). In the treprostinil-treated group, one animal had to be euthanized for bone marrow failure, but the overall survival curves did not differ in a statistically significant way (Fig. 3e). In the 38 animals, which had survived until day 15, blood cell counts (i.e., for erythrocytes, leukocytes, and platelets) were comparable and within the normal range (Fig. 3f–h).

### Histopathological consequences of treprostinil treatment

**Fig. 4:**
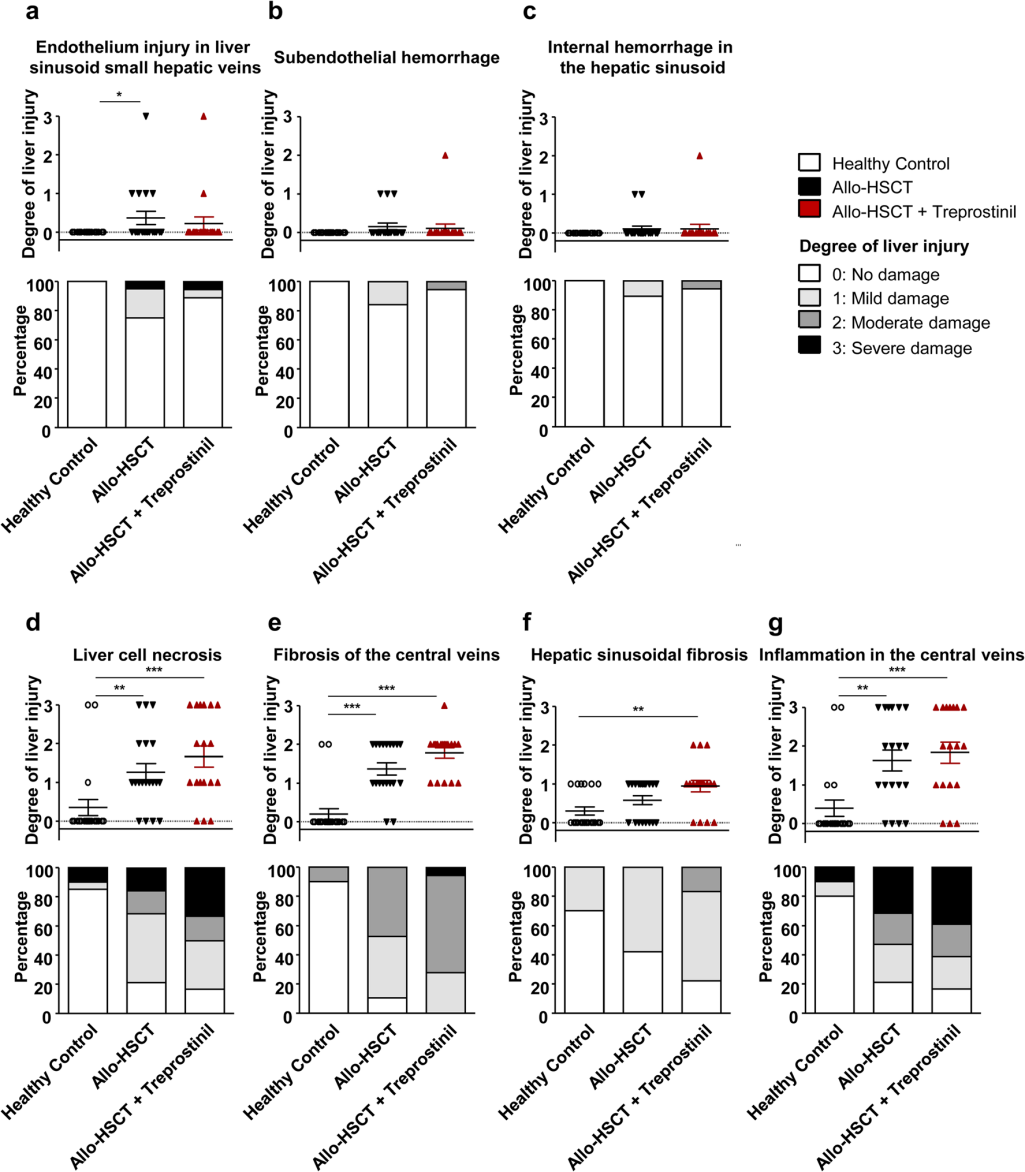
Histopathological analysis of murine liver sections provides evidence for reduced injury to LSECs and hemorrhage but increased inflammation and fibrosis upon treprostinil treatment. Preconditioned BALB/c mice (whole body irradiation of 7.5 Gy) received bone marrow cells from C57BL/6 mice. One set of mice was treated subcutaneously 1 day before and the subsequent 15 days after allo-HSCT with treprostinil (0.15 mg kg^−1^ 8 h^−1^). Based on H&E and Masson-Goldner trichrome stains, liver sections of experimental mice were blindly evaluated by a pathologist. The scoring system was adapted from Qiao et al. and Zeng et al. [27, 28]. Histological changes of seven liver damage scores were examined. Liver damage within zero, one, two, or three scores was classified into no, mild, moderate, or severe damage, respectively. Histopathological analysis indicated that treatment with treprostinil (0.15 mg kg^−1^ 8 h^−1^) led to reduced sinusoidal endothelial injury (*n* ≥ 18/group) but increased necrosis and fibrosis. **a** Endothelium injury in liver sinusoid or small hepatic veins. **b** Subendothelial hemorrhage. **c** Internal hemorrhage in the hepatic sinusoid. **d** Liver cell necrosis. **e** Fibrosis of the central veins. **f** Hepatic sinusoidal fibrosis. **g** Inflammation in the central veins. Each histological feature is represented as stacked bar graph (percentage distribution) and scatter plot (statistical evaluation) representing the degree of liver injury. Differences between groups were examined for their statistical significance by a Kruskal-Wallis test followed by Dunn’s post hoc test for multiple comparisons (*p < 0.05, **p < 0.01, ***p < 0.001)

**Fig. 5:**
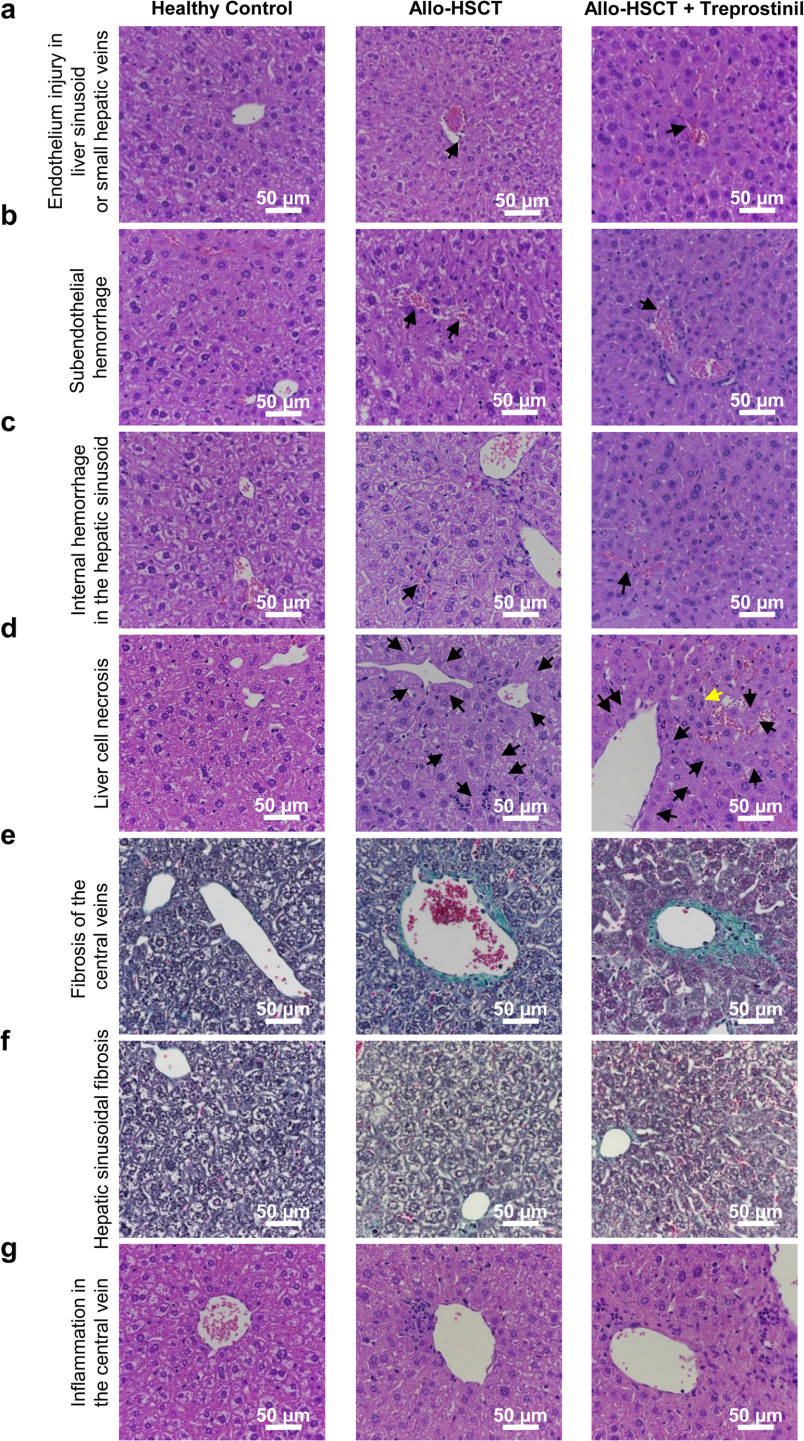
Histopathology of murine liver sections. Following whole body irradiation (7.5 Gy) and transplantation of bone marrow cells obtained from C57BL/6 mice, one group of BALB/c mice was treated subcutaneously 1 day before and the subsequent 15 days after allo-HSCT with treprostinil (0.15 mg kg^−1^ 8 h^−1^). Representative micrographs of liver section are shown as H&E or Masson-Goldner trichrome stains, respectively. **a** Sinusoidal endothelial injury (H&E) shows fibrin deposition in central vein and some destruction of endothelium in allo-HSCT mice (black arrow), as well as slight dilatation of sinusoids and red blood cell congestion in treprostinil-treated mice (black arrow). **b**, **c** Subendothelial hemorrhage and internal hemorrhage in the hepatic sinusoid (H&E) are indicated by black arrows. **d** Liver cells without remaining nuclei are indicative of liver cell necrosis in allo-HSCT and allo-HSCT + treprostinil mice (H&E, black arrows); a single shrinking apoptotic cell with nuclear pyknosis in treprostinil-treated mice is shown (H&E, yellow arrow). **e**, **f** As shown by Masson-Goldner trichrome staining of livers, fibrosis of central veins and hepatic sinusoidal fibrosis is present in both groups. Green: collagen, connective tissue. Orange-red: red blood cells. **g** Inflammation in the central veins in both groups (H&E)

The data summarized in Fig. 3 indicated that administration of treprostinil mitigated the development of one of the cardinal symptoms of SOS, i.e., hepatomegaly. However, treprostinil did not have a clear-cut effect on liver damage assessed by plasma levels of ALT. Histological sections of the liver were surveyed to explore the reason for this discrepancy. The changes were scored (0 to 3 points/item) along seven dimensions [27]; the resulting quantification is shown in Fig. 4. Representative micrographs are displayed in Fig. 5. As evident from this analysis that the administration of treprostinil reduced damage to the liver sinusoids, because endothelium injury (Figs. 4a and 5a), subendothelial hemorrhage (Figs. 4b and 5b), and internal hemorrhage in hepatic sinusoids (Figs. 4c and 5c) occurred more frequently in animals that had undergone allogeneic hematopoietic stem cell transplantation than in in treprostinil-treated recipient mice. However, liver cell necrosis was not prevented but rather aggravated in treprostinil-treated recipient mice (Fig. 4d). Consistent with the increased vulnerability of zone 3, the liver cell necrosis was most pronounced at the center of the liver acini but also was associated with inflammation and hemorrhage (Fig. 5d). Similarly, treatment with treprostinil enhanced the inflammatory response surrounding the central veins (Figs. 4g and 5g). Finally, the fibrosis detected by Masson-Goldner trichrome staining was most frequently seen in treprostinil-treated recipient mice in both the hepatic sinusoids (Figs. 4f and 5f) and the area surrounding the hepatic central veins (Figs. 4e and 5e). In our blinded assessment, we also detected cell necrosis of the liver as well as signs of inflammation and fibrosis in healthy BALB/C mice not subjected to any treatment (Fig. 4d–g). The reason for this preexisting pathology is not clear. However, we stress that we verified that the animals did not carry any specific pathogens; in particular, they were free of mouse hepatitis virus.

## Discussion

SOS is a challenging disease from the perspective of both basic research and clinical management. Toxic injury to liver sinusoidal cells is thought to be the central pathogenic event that occurs in the early phases after HSCT. We employed a murine SOS model, which is known to peak on day 15 and to subsequently resolve spontaneously [27]. Accordingly, we selected day 15 to assess the extent of liver damage. Our observations recapitulated the original findings; this is most readily evident by considering the extent of hepatomegaly and the elevations of ALT levels: our values are in excellent agreement with those reported by Zeng et al. [27]. Similarly and consistent with the observations, we also found that changes in bilirubin levels were less useful to monitor hepatic damage. This is not unexpected because ablation of the bone marrow and the subsequent reconstitution of the bone marrow by hematopoietic stem cell transplantation are likely to affect red blood cell dynamics and thus the production of bilirubin. Our study was powered to detect a beneficial effect of treprostinil provided that it resulted in a reduction in liver weight. Based on hepatomegaly as primary outcome parameter, the treatment of recipient mice with treprostinil was indeed effective in mitigating SOS.

Our working hypothesis posited that treprostinil acted via G_s_-coupled prostanoid receptors on the LSECs. We verified the presence of transcripts of *Ep*_2_, *Ep*_4_, and *Ip* receptors in LSECs. Similarly, the histological analysis suggested that treprostinil reduced injury to liver sinusoidal endothelial cells. The effect, which we detected in the histopathology, was modest, presumably because our analysis was limited to a snapshot on day 15, i.e., long after the initial injury. More importantly, the histological analysis revealed a damaging effect of treprostinil on the hepatocytes. This is surprising, because prostaglandins are thought to be beneficial in liver injury and their involvement in regeneration is complex: PGE_2_ and to a lesser extent PGI_2_ stimulate hepatocyte proliferation [30]; the effect is relevant under in vivo conditions, because genetic ablation of the prostaglandin degrading enzyme 15-hydroxyprostaglandin dehydrogenase (15-PGDH) or its inhibition by a small molecule enhances liver regeneration after partial hepatectomy [31]. Conversely, liver regeneration after surgical removal of liver tissue is blunted upon inhibition of cyclooxygenase-2 and to a lesser extent of cycloxygenase-1, the enzymes that catalyze the conversion of arachidonic acid to prostaglandin H2, the precursor of PGE_2_, PGI_2_, and other prostaglandins [32]. These findings suggest a role of endogenous PGE_2_ in promoting hepatocyte regeneration. It is less clear which prostanoid receptors mediate the beneficial actions of PGE_2_: originally, G_q_-coupled E prostanoid receptors were shown to be more important for stimulating the proliferation of hepatocytes than G_s_-coupled receptors [30], but in ischemia-reperfusion injury, the protective effect was conveyed by stimulation of the EP_4_ receptor [33]. However, more recently, the beneficial effects of PGE_2_ and in particular of the EP_4_ receptor have been questioned [34]: in fact, inhibition of PGE_2_ synthesis—by deletion of the inducible microsomal PGE synthase-1 or by its inhibition with a small molecule—mitigated liver cell necrosis resulting from ischemia and reperfusion. Similarly, blockage of the EP_4_ receptor—not of the EP_1_ or of the EP_2_ receptor—protected against hepatocyte damage and reduced necrotic areas in mice, in which the EP_4_ receptor was absent due to genetic deletion [34]. Thus, the available evidence shows that the activation of G_s_-coupled prostaglandin receptors results in both enhanced and reduced hepatocyte necrosis depending on the nature of the injury and the time of the snapshot. In fact, the conflicting results of Kuzumoto et al. [33] and of Nishizawa et al. [34] can be rationalized by considering that the time points sampled differed: Kuzumoto et al. [33] examined early events (i.e., 2 h after reperfusion), while the observation period of Nishizawa et al. [34] focused on changes occurring days after ischemia and reperfusion. The pathophysiology underlying damage caused by SOS is presumably more akin to that of ischemia and reperfusion than that of partial hepatectomy. Furthermore, our analysis focused on late events, which are presumably more relevant for the clinical treatment of SOS. Taken together, the previously published findings and our current observations indicate that treprostinil is a double-edged sword. While its action on the LSECs and on platelets may be beneficial, its action on macrophages/Kupffer cells and/or hepatocytes limits the usefulness in the treatment of SOS. This is presumably also true for other agonists, which target G_s_-coupled prostanoid receptors.

Defibrotide is the only drug which is currently available for the treatment of SOS [4, 5]. The approval of defibrotide is based, in part, on a phase III trial, which—for ethical reasons—relied on a carefully selected historical control group [35]. Based on this trial, it is clear that the unmet medical need is large: the numbers needed to treat were 5 and 6 for preventing death within 100 days and 6 for a complete response (i.e., a reduction in serum bilirubin < 2 mg/100 mL, creatinine clearance > 80% of initial value, and oxygen saturation > 90% with ambient air), respectively. It is evident that there is room for improvement. In contrast to treprostinil, which is a chemically defined small molecule, defibrotide is a polydisperse mixture of oligonucleotides obtained from porcine intestinal mucosa. The heterogeneity of defibrotide hampers progress, because it is not clear which of its many reported actions are relevant to improve the outcome in SOS. Administration of defibrotide is associated with a substantial risk of hemorrhage (i.e., affecting 5 to 10% of the patients) and of hypotension. Finally, data on the long-term safety of defibrotide are not yet available. The advantage of treprostinil is not only its well-defined mechanism of action but also the fact that its safety profile is well understood in both adults [18–20] and children [18–20]. However, treprostinil can only be considered a viable candidate drug for SOS, if it shows convincing efficacy in a credible preclinical model.

We stress that we used a dose of 0.45 mg kg^−1^ day^−1^ treprostinil, which—after correction for allometry—corresponds to the clinical dose in man: it is the dose which can be safely administered in the treatment of pulmonary hypertension and shows maximum efficacy [18–20]. In fact, this dose provided the optimum beneficial effect in promoting hematopoietic stem cell transplantation in lethally irradiated recipient mice [24]. In previous work, we noted a bell-shaped dose-response curve of treprostinil, suggesting that a higher dose of treprostinil is less beneficial than the currently used dose. We cannot formally exclude that a variation in treprostinil dose would have an uncovered window, where the beneficial effect would have outweighed the detrimental action. However, we consider it unlikely that this can be detected with an ethically justifiable number of experimental animals because of the large inherent variability in the course of SOS. Currently, prostaglandins such as PGE_1_ are not recommended for the treatment of SOS [35], because an encouraging initial trial [15] was not replicated [16]. At the very least, our observations provide an explanation for this failure: in our murine SOS model, stimulation of G_s_-coupled was protective for the endothelium of the liver sinusoids, but we also detected a damaging effect of treprostinil on hepatocytes. This detrimental action is likely to limit or cancel out any benefit arising from the administration of prostaglandins in human SOS.

## Abbreviations

SOS: Sinusoidal obstruction syndrome
HSCT: Hematopoietic stem cell transplantation
ALT: Alanine aminotransferase
LSEC: Liver sinusoidal endothelial cells
PGI_2_: Prostacyclin
PGE_2_: Prostaglandin E_2_
cAMP: Cyclic adenosine monophosphate
IP: I prostanoid
EP: E prostanoid
Allo-HSCT: Allogeneic stem cell transplantation
18S: 18S ribosomal RNA
qPCR: Quantitative PCR
H&E: Hematoxylin-eosin
WBC: White blood cell
RBC: Red blood cell
PLT: Platelet
LW/BW: Liver weight to body weight
